# Spinal cord stimulation for concurrent improvement of consciousness and spasticity following severe traumatic brain injury: a case study

**DOI:** 10.3389/fnins.2026.1866882

**Published:** 2026-07-28

**Authors:** Yitong Jia, Jianming Wang, Tan Zhang, Sipeng Zhu, Nan Wang, Qiheng He, Tianqing Cao, Xiaoke Chai, Yiyi Zhai, Yi Yang

**Affiliations:** 1 Department of Neurosurgery, Beijing Tiantan Hospital, Capital Medical University, Beijing, China; 2 China National Clinical Research Center for Neurological Diseases, Beijing, China; 3 National Key Laboratory of Advanced Micro and Nano Manufacture Technology, School of Integrated Circuits, Peking University, Beijing, China; 4 Department of Neurosurgery, The Second Affiliated Hospital of Soochow University, Jiangsu, China

**Keywords:** consciousness-motor circuit, disorders of consciousness, neuromodulation, spasticity, spinal cord stimulation

## Abstract

**Background:**

Spasticity frequently complicates disorders of consciousness (DoC) after severe brain injury, yet effective treatments addressing both conditions remain limited. Spinal cord stimulation (SCS) has shown promise in treating each condition independently, but its application in patients with concurrent DoC and spasticity has rarely been systematically investigated.

**Methods:**

We report a 30-year-old man with prolonged DoC and severe limb spasticity following traumatic brain injury who underwent dual-level percutaneous SCS lead implantation. Frequency screening was performed to identify optimal stimulation parameters, followed by 21 days of continuous stimulation. Assessments included the Coma Recovery Scale–Revised (CRS-R), Modified Ashworth Scale (MAS), Hip Adductor Tone Scale (HATS), surface electromyography (sEMG), electroencephalography (EEG), and transcranial magnetic stimulation (TMS).

**Results:**

Frequency screening identified 80 Hz as the optimal parameter for spasticity reduction. After 21 days of stimulation, the CRS-R score increased from 5 to 10, MAS improved from 3–4 to 1–1+ , and HATS decreased from 3 to 1. sEMG showed reduced muscle activity and spectral shifts consistent with decreased spasticity. EEG demonstrated enhanced frontotemporal functional connectivity and increased spectral power. TMS motor evoked potential latency was significantly shortened. Clinical improvements were sustained at the 6-month follow-up.

**Conclusion:**

This case suggests that 80 Hz SCS may be associated with concurrent improvement in consciousness and spasticity in patients with DoC following severe brain injury, accompanied by electrophysiological correlates of altered neural connectivity and corticospinal function.

## 1 Introduction

Disorders of consciousness (DoC) are characterized by impaired arousal and awareness following severe brain injury (Edlow et al., 2021). Spasticity is a frequent complication in DoC, affecting approximately 59%–89% of patients (Nakase-Richardson et al., 2013; Thibaut et al., 2015; Zhang et al., 2021). It mainly results from an exaggerated stretch reflex driven by a fundamental imbalance between spinal excitability and its supraspinal regulation (Kheder and Nair, 2012). Owing to severe and widespread brain damage, patients with DoC exhibit atypical clinical patterns and generally develop more severe spasticity than individuals with isolated stroke or spinal cord injury (Martens et al., 2017). Moreover, the inability of DoC patients to perceive or communicate spasticity-related pain further aggravates muscle hypertonia, creating a self-perpetuating vicious cycle (Thibaut et al., 2015).

Spinal cord stimulation (SCS) has shown partial efficacy in treating DoC and spasticity independently (Powell et al., 2023; Jung et al., 2024; Sun et al., 2024; Zhuang et al., 2024; Asp et al., 2025; Ren et al., 2025; Romeni et al., 2025). However, the technical implementation of SCS differs between these two indications. In patients with DoC, high cervical stimulation has been used to engage arousal related ascending pathways, and short term SCS protocols have increasingly adopted percutaneous multi-contact cylindrical leads to reduce procedural invasiveness in this medically fragile population (Zhuang et al., 2022; He et al., 2024). By contrast, spasticity oriented SCS places greater emphasis on stable, segment specific recruitment of spinal sensorimotor circuits, because stimulation effects are strongly influenced by electrode location, contact configuration, and targeted spinal roots (Blackburn et al., 2021; Greiner et al., 2021). Nevertheless, systematic investigations into SCS for patients with DoC complicated by spasticity remained limited and are largely confined to the work of Mayorova and colleagues, which focused predominantly on hypothesizing the therapeutic mechanisms of SCS in this clinical context (Vorobyev et al., 2023, 2024; Mayorova L. et al., 2025; Mayorova L. A. et al., 2025). These studies were methodologically constrained, characterized by insufficient clinical description and reproducible protocols.

We conducted a series of observations and investigations in a patient with severe traumatic brain injury (TBI) who presented with DoC complicated with limb spasticity and underwent implantation of SCS. We reported detailed procedures encompassing electrode implantation, stimulation frequency screening, quantitative assessments, and longitudinal follow-up. The aim was to characterize the coupled changes in spasticity and arousal-related measures following SCS and provide novel insights into the clinical management of this patient population. This case report has been reported in line with the SCARE checklist (Kerwan et al., 2025).

## 2 Methods

### 2.1 Data availability

The raw data are available from the corresponding author upon reasonable request.

### 2.2 Study design

**FIGURE 1:**
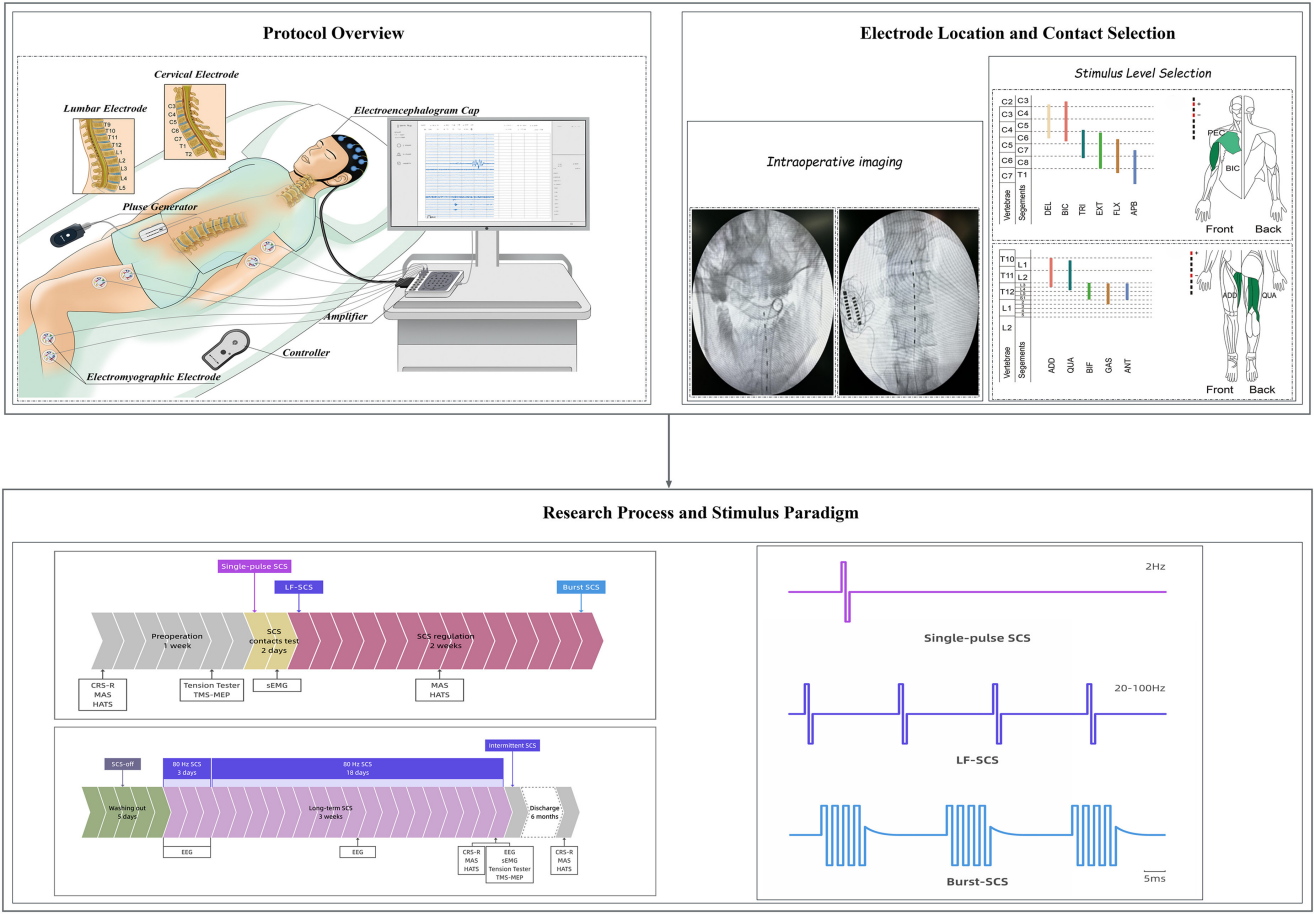
Overview of the study workflow.

This was a single-center retrospective medical record study at Beijing Tiantan Hospital. The study was conducted in accordance with the principles of the Declaration of Helsinki. The protocol was approved by the Ethics Committee of Beijing Tiantan Hospital. Written informed consent was obtained from the patient’s legal surrogate. The overall clinical and assessment workflow was illustrated in Figure 1.

### 2.3 Case presentation

A 30-year-old man was admitted 5 months after neurosurgical treatment for severe TBI. Initial emergency CT following the trauma revealed multiple cerebral contusions and lacerations with intracranial hematoma and signs of brain herniation. The patient underwent emergent hematoma evacuation and decompressive craniectomy, after which he was transferred to the intensive care unit for further management.

One week after injury, the patient exhibited spontaneous eye opening without any purposeful response to external stimuli, consistent with a severe DoC. At approximately 1-month post-injury, marked flexion and adduction spasticity developed in the right upper and lower extremities.

During the period from surgery to admission to our institution, the patient experienced symptomatic seizures and pneumonia but no other life-threatening acute conditions, including metabolic disturbances or active infection. The tracheostomy was successfully decannulated. Prior treatments included levetiracetam for seizure control and cephalosporin antibiotics for infection. Amantadine was administered to promote arousal, and oral baclofen was used for spasticity management in combination with physical rehabilitation therapy. However, neither the level of consciousness nor the severity of spasticity demonstrated substantial improvement. Prior clinical studies and the routine practice at our center have applied SCS for both spasticity management and arousal facilitation. Accordingly, SCS was considered a therapeutic option to address the coexisting impaired consciousness complicated with severe spasticity in this patient.

### 2.4 Preoperative planning

#### 2.4.1 Clinical assessments

**FIGURE 2:**
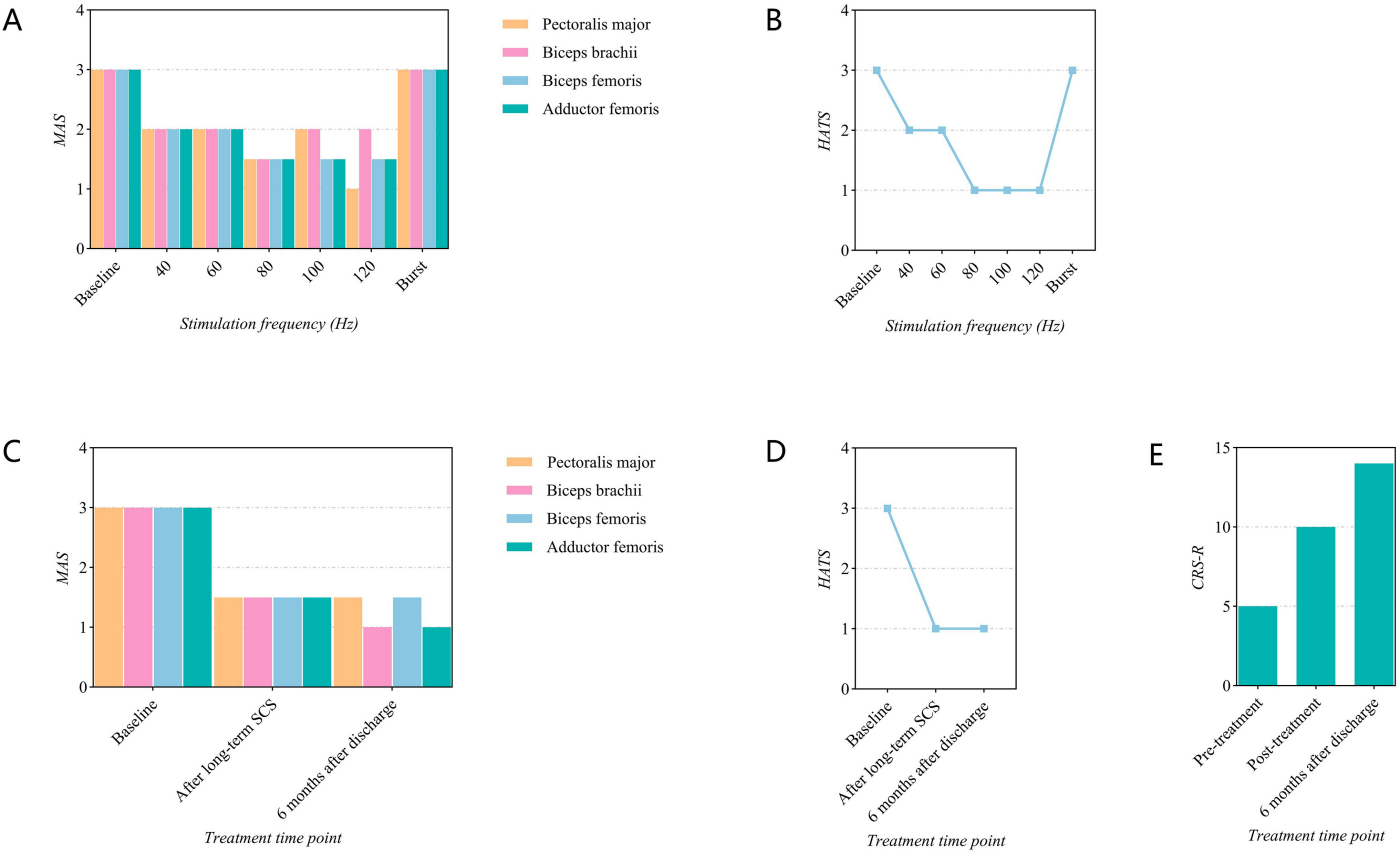
Changes in clinical scores before and after SCS. **(A)** MAS scores of the four predefined target muscle groups at baseline and after 2-day exposure to each screening condition, including 40, 60, 80, 100, and 120 Hz tonic SCS and burst-mode stimulation. MAS 1 + was plotted as 1.5 for visualization. **(B)** HATS scores at baseline and after 2-day exposure to each screening condition. **(C)** MAS scores at baseline, after completion of 21-day continuous 80 Hz SCS, and at 6 months after discharge. **(D)** HATS scores at baseline, after completion of 21-day continuous 80 Hz SCS, and at 6 months after discharge. **(E)** CRS-R scores at pretreatment baseline, after completion of 21-day continuous 80 Hz SCS, and at 6 months after discharge.

Preoperative consciousness assessments were conducted using Coma Recovery Scale–Revised (CRS-R), which yielded a score of 5 points indicating vegetative state (Figure 2 and Supplementary Video). Modified Ashworth scale (MAS) for right shoulder adductors, elbow flexors, wrist flexors, adductor femoris, and knee flexors were all ranked as 3, whereas the shoulder flexors and extensors were grade 2 (Figure 2 and Supplementary Video). Both ankle joints muscles were rated as level 4. For the hip adductors tension scale (HATS), it was 3 points on the right side and 0 points contralaterally (Figure 2). The duration of spasticity was usually 10–12 h per day. All left limbs had a MAS score of almost 0, without focusing on muscles with MAS < 2.

#### 2.4.2 Assessment of residual corticospinal pathway

Given that post-injury CT demonstrated damage involving the motor cortex and bilateral basal ganglia, transcranial magnetic stimulation (TMS) was used to evaluate corticospinal tract function. The patient was placed in a supine position. Stimulation was delivered using a figure-of-eight coil connected to a magnetic stimulator system (NS 1000, Wuhan Yiruide Medical Equipment New Technology Co., Ltd., Wuhan, China) over the scalp projection of the left primary motor cortex corresponding to the hand area. Motor evoked potentials (MEPs) were recorded from the right abductor pollicis brevis (APB) using TMS-associated surface electromyography (TMS-sEMG), which was acquired through the integrated electromyographic recording module and acquisition software of the TMS system, without a separate sEMG device model.

For the hand area, the coil was oriented approximately 45° toward the contralateral forehead to induce a current roughly perpendicular to the central sulcus. Resting motor threshold (RMT) was defined as the minimum stimulation intensity capable of eliciting reliable MEPs of minimal amplitude in the target muscle. According to the recommendations of Rossini et al. (2015) stimulation began at 35% of maximum stimulator output (MSO) and was increased in 5% MSO increments until MEPs with a peak-to-peak amplitude ≥50 μV were consistently evoked in 10 trials. The intensity was then decreased in 1% MSO steps until fewer than 5 positive responses were observed in 10 trials. RMT was defined as 1% MSO above this level.

Pre-SCS TMS-MEP assessment was performed before the initial temporary SCS lead implantation. At this assessment, RMT was 41% of MSO. After seven repeated stimuli, the mean latency of MEP recorded from right APB was 50.6 ± 24.9 ms, with a mean peak-to-peak amplitude of 105.6 ± 16.0 μV (Table 1). Post-SCS TMS-MEP assessment was performed after completion of the 3-week stimulation period following implantable pulse generator implantation. All procedures were performed by an experienced neurorehabilitation physician.

**TABLE 1:** TMS-MEP latency and amplitude before SCS and after completion of the 3-week stimulation period.

Trial no.	Latency^a^		Amplitude^b^		Mean ± SD				*P*-value	
	Pre-SCS	Post-SCS	Pre-SCS	Post-SCS	Latency period		Amplitude		Latency period	Amplitude
					Pre-SCS	Post-SCS	Pre-SCS	Post-SCS		
1	31.6	24.0	124.0	128.5	50.6 ± 24.9	19.6 ± 3.7	105.6 ± 16.0	130.3 ± 26.6	0.007	0.054
2	63.6	16.0	114.0	103.8						
3	31.2	14.0	89.0	100.5						
4	19.4	22.4	102.0	180.6						
5	70.6	21.5	92.0	130.0						
6	49.4	21.5	124.0	140.8						
7	88.4	17.5	94.0	127.6						

SCS, spinal cord stimulation.

*^a^*Unit of measurement: ms.

*^b^*Unit of measurement: μV.

### 2.5 Electrodes implantation

A temporary percutaneous SCS trial system (PINS) was first implanted. Given the patient’s prolonged bed-bound status and reduced physiological reserve after long-term immobilization, percutaneous 8-contact leads were implanted at both the cervical and thoracolumbar levels to preserve the procedural advantages of percutaneous stimulation while targeting both impaired consciousness and unilateral limb spasticity. Two 8-contact cylindrical leads with 4-mm intercontact spacing (model L3213) were used. The externalized end of each lead was connected to a test cable (A6216), which was then coupled to the external pulse generator (T902).

The patient was placed in the prone position under general anesthesia. Percutaneous epidural access was obtained at the T7 level, approximately aligned with the inferior angle of the scapula, using a Tuohy needle. An 8-contact cylindrical lead was then advanced under fluoroscopic guidance. Based on previously reported relationships between the lateralization of affected muscle groups and the corresponding segmental innervation of the spinal cord, two epidural leads were implanted along the dorsal root entry zone slightly to the right of the midline (Figure 1; Schirmer et al., 2011). The cervical lead was positioned longitudinally from the C2 to C6 vertebral levels, whereas the thoracolumbar lead extended from T11 to L1. Later intraoperative testing was performed via a temporary pulse generator connected to the lead. Stimulation was delivered at 2 Hz with a pulse width of 210 μs, and the current amplitude was increased in 0.1 mA increments to elicit motor responses. SCS-associated surface electromyography (SCS-sEMG) recordings were acquired using an Aci-EM electrophysiological recording system (Guangzhou Ximang Medical Technology Co., Ltd., Guangzhou, China) with an Aci-EM32 amplifier recording box. SCS-sEMG recordings demonstrated stimulation-evoked potentials in different target muscle groups, confirming appropriate electrode positioning.

The externalized lead was subsequently secured. The procedure was performed by an experienced neurosurgeon and required approximately 15 min. Postoperative cervical and thoracolumbar CT imaging confirmed lead placement and excluded procedure related complications (Figure 1).

### 2.6 Postoperative regulation and evaluation

#### 2.6.1 Contact selection

Before stimulation, we first assessed the muscle recruitment capacity of different contact configurations. As the patient mainly presented with severe right elbow flexion, wrist flexion, and knee flexion, contact selection focused primarily on these three muscle groups. Bipolar single-pulse stimulation was applied, with each contact tested as both the anode and cathode, at 2 Hz and 210 μs. For each contact pair, stimulation intensity was increased in 0.1 mA increments until stable MEPs were identified on sEMG and the peak-to-peak amplitude reached a plateau. The mean peak-to-peak amplitude of each target muscle was recorded at each intensity level, and normalized peak-to-peak amplitudes were calculated according to Powell et al. (2023) as the ratio of the mean amplitude at a given intensity to the maximal amplitude recorded for that muscle across all contact configurations and current intensities. sEMG signals were band-pass filtered at 20–450 Hz with an additional 48–52 Hz notch filter. In the cervical lead, the 2 + 4- configuration produced the strongest recruitment of the right biceps brachii. Effective activation of the right wrist flexors required higher stimulation intensities, which also caused undesirable recruitment of non-spastic muscles. In the right lower limb, the 1 + 5- configuration on the thoracolumbar lead elicited the greatest recruitment of the right biceps femoris and adductor muscles.

#### 2.6.2 Regulatory period

The patient subsequently underwent tonic SCS testing with frequency increments of 20 Hz, covering a range from 40 to 120 Hz, and burst mode was ultimately explored. For each tested frequency, the stimulation amplitude was adjusted in 0.1 mA increments while maintaining a constant pulse width of 210 μs. The optimal amplitude at a given frequency was defined as the lowest intensity that elicited mild involuntary muscle activation without inducing observable signs of discomfort, such as grimacing or startle responses. The stimulation was administered daily from 8:00 AM to 8:00 PM, totaling 12 h per day. Two days following stimulation at each frequency, MAS and HATS scores were evaluated for four passive movements: shoulder abduction, elbow extension, knee extension, and hip abduction. The spastic muscle groups involved in each passive movement were designated as target muscles for scoring. These four movements correspond to pectoralis major, biceps brachii, biceps femoris, and adductor femoris, respectively.

Upon completion of frequency screening, the optimal stimulation frequency was selected based on a combined assessment of efficacy and tolerability. Efficacy was primarily determined by the overall reduction in MAS scores across the four target muscle groups and HATS scores after 2-day exposure to each frequency, rather than by the lowest score in a single muscle. Particular attention was paid to whether clinically relevant residual tone persisted in key target muscles, especially the biceps brachii, because elbow flexor spasticity is closely related to upper-limb activity limitation, contracture development, hygiene, dressing, and caregiver burden (Ada et al., 2006; Sheean et al., 2010). In clinical spasticity studies, MAS ≥ 2 is commonly used to indicate clinically relevant hypertonia requiring intervention (Elovic et al., 2016). Tolerability was assessed according to observable discomfort or autonomic/adverse responses during stimulation. Given that this was an exploratory single-case sequential screening protocol, potential carry-over effects between frequencies could not be completely excluded. However, the selected frequency was further evaluated after a 5-day washout period during subsequent continuous stimulation.

Subsequently, the temporary SCS system was removed, and an implantable pulse generator (G122R) was subcutaneously implanted in the lumbosacral region.

#### 2.6.3 Electrophysiological examination

##### 2.6.3.1 SCS-sEMG recordings

We assessed the long-term effects of continuous SCS using electroencephalography (EEG) and SCS-sEMG. EEG and SCS-sEMG signals were acquired using the same Aci-EM digital EEG/sEMG recording system and Aci-EM32 amplifier recording box (Guangzhou Ximang Medical Technology Co., Ltd., Guangzhou, China). To minimize carry-over effects, permanent SCS was initially discontinued for 5 days before applying 80 Hz stimulation for 3 weeks.

Spinal cord stimulation- associated surface electromyography recordings were obtained 2–3 times daily during the first 3 days of stimulation, with 2-h intervals between sessions and no nocturnal measurements. Each session involved rapid passive stretching of four target muscle groups. SCS-sEMG signals were preprocessed using the same filtering procedure described above, including band-pass filtering at 20–450 Hz and notch filtering at 48–52 Hz to reduce power-line interference.

##### 2.6.3.2 EEG acquisition and preprocessing

Electroencephalography recordings were obtained at four time points: E0 (pre-SCS baseline), E1 (day 1), E2 (day 10), and E3 (day 21). Each EEG recording session lasted more than 10 min under resting state conditions with stimulation turned off. During EEG acquisition, the patient was placed in the supine position, and efforts were made to minimize movement throughout the recording. Recordings were conducted in a quiet environment under standardized lighting conditions and were continuously monitored by the same clinical investigator. If the patient showed signs of sleep, indicated by prolonged eye closure or the appearance of sleep spindles or K-complexes on EEG, the session was paused, and the JFK CRS-R arousal facilitation protocol was administered to restore arousal.

Electroencephalography was recorded using a 20-scalp-channel montage based on the international 10–20 electrode placement system, with bilateral mastoid electrodes used for referencing. The 20 scalp electrode sites were Fp1, Fp2, F7, F3, Fz, F4, F8, T3, C3, Cz, C4, T4, T5, P3, Pz, P4, T6, O1, Oz, and O2. After harmonization of channel names, the first 10 s of each recording were discarded. The raw data were first notch filtered at 48–50 Hz to attenuate line noise contamination from the power supply and then band pass filtered at 0.5–45 Hz. The 0.5 Hz high pass cutoff was used to reduce slow baseline drift and ultra-low frequency artifacts, whereas the 45 Hz low pass cutoff was applied to suppress high frequency noise, including muscle related interference.

Bad channels were automatically detected and interpolated based on amplitude- and correlation-based criteria. Artifact contaminated segments were automatically detected using a sliding window amplitude threshold of ± 100 μV. Only clean epochs were retained and concatenated for subsequent analysis.

##### 2.6.3.3 EEG spectral and functional connectivity analysis

Given that both regional neural activity and interregional synchrony are relevant to consciousness, power spectral density (PSD) and phase-locking value (PLV) were calculated after EEG preprocessing to quantify frequency-specific neural activity and functional connectivity, respectively (Lachaux et al., 1999; Alnes et al., 2025). PSD was used to characterize oscillatory power across canonical frequency bands, whereas PLV was used to estimate phase synchronization between EEG signals, reflecting the degree of neural coupling between channels or regions.

Power spectral density features were extracted from the preprocessed resting-state EEG recordings using custom Python scripts. For each recording, EEG signals were converted to microvolts before spectral estimation. PSD was calculated using Welch’s averaged periodogram method with Hann-windowed 2-s segments and 1-s overlap, within the frequency range of 0.5–40 Hz. Spectral power was then summarized within six canonical frequency bands: delta, 0.5–4 Hz; theta, 4–8 Hz; alpha, 8–13 Hz; beta 1, 13–20 Hz; beta 2, 20–30 Hz; and gamma, 30–40 Hz.

For PLV analysis, EEG signals were first decomposed into the same frequency bands. For each frequency band, the instantaneous phase of each channel was extracted, and PLV was calculated for each pair of channels based on the consistency of phase differences over time. Higher PLV values indicate stronger phase synchronization between two EEG signals (Lachaux et al., 1999). PLV values were calculated separately for each frequency band. For regional analysis, EEG channels were partitioned according to the international 10–20 system and anatomical location. Considering the predominantly left sided structural injury and the potential involvement of midline networks after traumatic diffuse axonal injury, the regional analysis was restricted to left hemispheric and midline channels. The 12 channels included in the regional analysis were assigned *a priori* to five regions: frontal, F: Fp1, F3, F7, and Fz; central, C: C3 and Cz; temporal, T: T3 and T5; parietal, P: P3 and Pz; and occipital, O: O1 and Oz. Average PSD (APSD) was defined as the mean PSD across channels within each predefined region. Given the relevance of frontal networks to consciousness, frontal-regional PLV was calculated as the mean PLV between all channels in the frontal region and all channels in each target region.

Based on these region averaged metrics, band-specific temporal trajectories of APSD and PLV were first summarized across the four recording time points. Stimulation induced changes were then quantified as ΔAPSD and ΔPLV, calculated as E3 minus E0. For each frequency band, interregional differences in ΔAPSD and ΔPLV were compared across the predefined regions of interest. Non-parametric Kruskal–Wallis tests were used for exploratory interregional comparisons, followed by *post hoc* pairwise comparisons with false discovery rate correction. Pairwise regional contrasts were further calculated to characterize the proportional contribution of interregional differences. For APSD, each contrast was defined as the difference between frontal ΔAPSD and ΔAPSD in each non-frontal region. For PLV, each contrast was defined as the difference between two frontal-regional ΔPLV connections. The absolute magnitude of each contrast was expressed as a proportion of the summed absolute contrasts within the corresponding frequency band. A two-sided false discovery rate value <0.05 was considered statistically significant.

Following completion of the stimulation protocol, TMS-MEP was reassessed, together with the corresponding evaluations of consciousness and motor function.

The patient and family demonstrated excellent compliance throughout the perioperative period, with all procedures and evaluations completed smoothly and no reported discomfort.

## 3 Results

### 3.1 Regulation and clinical assessments

During frequency screening, 80 Hz tonic stimulation produced the most balanced reduction in muscle tone across the predefined target muscles. After 2-day exposure to 80 Hz stimulation, MAS scores decreased to 1 + in all four target muscle groups, and HATS decreased to 1 point (Figures 2A, B and Supplementary Video). Although 120 Hz stimulation resulted in a lower MAS score in the pectoralis major, the biceps brachii remained at MAS 2, indicating clinically relevant residual elbow flexor hypertonia (Elovic et al., 2016). Given the importance of elbow flexor tone for upper limb rehabilitation and the association between elbow flexor spasticity and contracture development, 120 Hz was considered less favorable than 80 Hz (Ada et al., 2006; Sheean et al., 2010). Burst mode stimulation was not selected considering that it was associated with adverse responses, including sweating and crying. Therefore, 80 Hz was chosen as the individualized optimal frequency based on the best overall balance between spasticity reduction across target muscles and stimulation tolerability.

### 3.2 sEMG assessments

**FIGURE 3:**
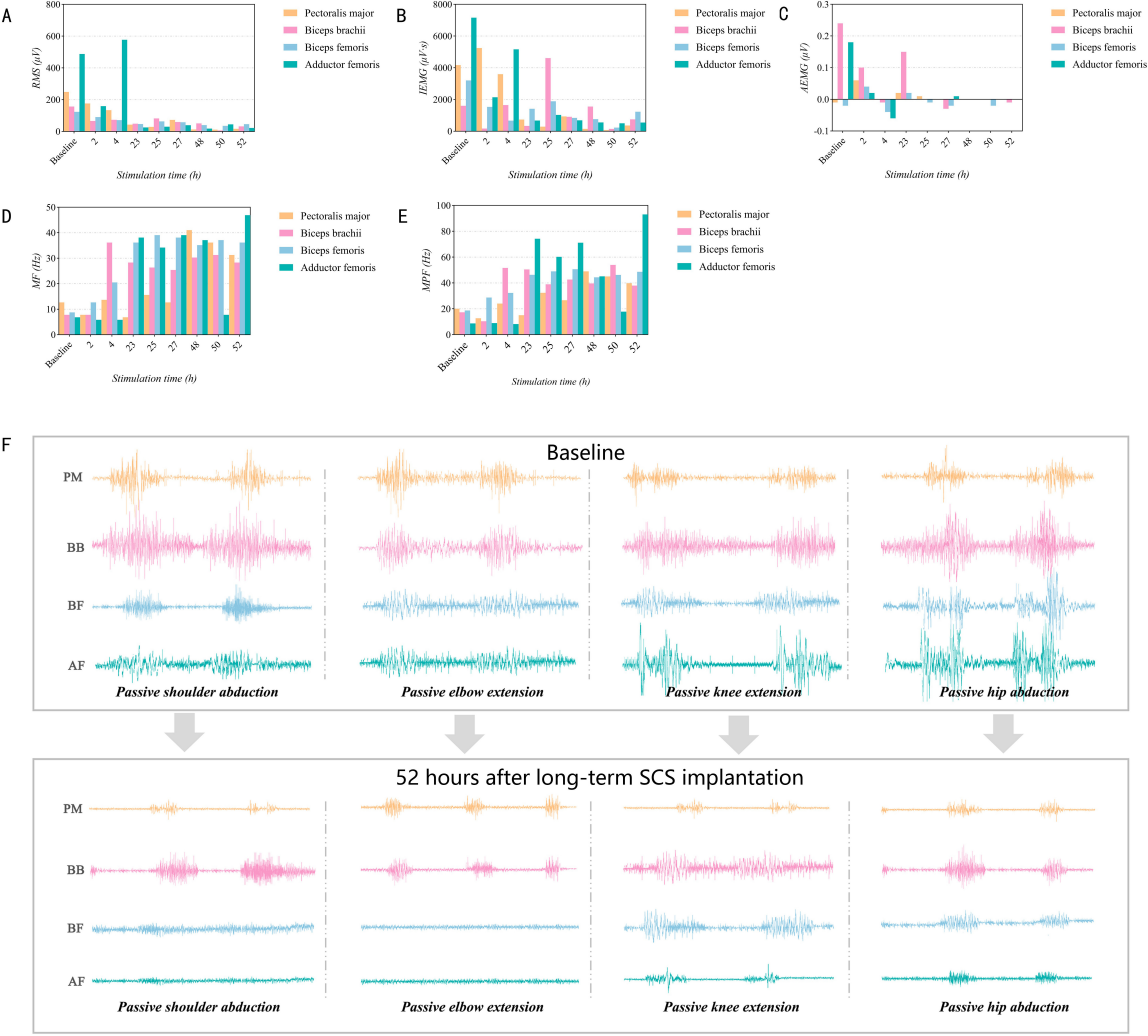
Surface electromyography (sEMG) metrics and representative waveforms before and during 80 Hz SCS. **(A–C)** Time-domain sEMG features of the four target muscles during passive movements, including root mean square (RMS), integrated electromyography (IEMG), and average electromyography (AEMG). **(D,E)** Frequency-domain sEMG features, including mean frequency (MF) and mean power frequency (MPF). **(F)** Representative sEMG waveforms recorded during passive shoulder abduction, elbow extension, knee extension, and hip abduction at baseline and 52 h after long-term SCS implantation. PM, pectoralis major; BB, biceps brachii; BF, biceps femoris; AF, adductor femoris.

At the end of 5 days washout period, MAS and HATS nearly returned to baseline levels. We found that sEMG time-domain indicators of targeted muscles, involving root mean square, average EMG (AEMG), integrated EMG (IEMG) exhibited remarkable decreases under 80 Hz SCS. For frequency domains, both mean frequency and mean power frequency showed a significant right shift phenomenon (Figure 3). The IEMG of the adductor femoris illustrated the dropping of 6606.82 μV following the first 3 days of SCS, while the MPF increased by 84.31 Hz.

Surface electromyography revealed decreased signal amplitude in spastic muscles during passive movement (Figure 3). Before stimulation, passive movement of either the upper or lower limb triggered cross limb spastic responses. After 52 h of stimulation, upper limb passive movement was associated with a more pronounced reduction in lower limb spasticity, whereas the reverse effect was less evident (Figure 3).

### 3.3 EEG assessments

The results demonstrated dynamic changes in both PLV and APSD following stimulation. PLV analysis revealed significant increases across all brain regions except the left frontal lobe on day 1 compared to baseline, with left hemispheric networks showing progressive enhancement by days 10 and 21, particularly in frontal, temporal, and parietal connectivity (Supplementary Figure 1). Concurrently, APSD analysis showed upward-shifting curves in most channels over time, indicating sustained elevation in spectral power that peaked on day 21, consistent with the observed heatmap patterns across primary frequency bands (Supplementary Figure 2, 3).

**FIGURE 4:**
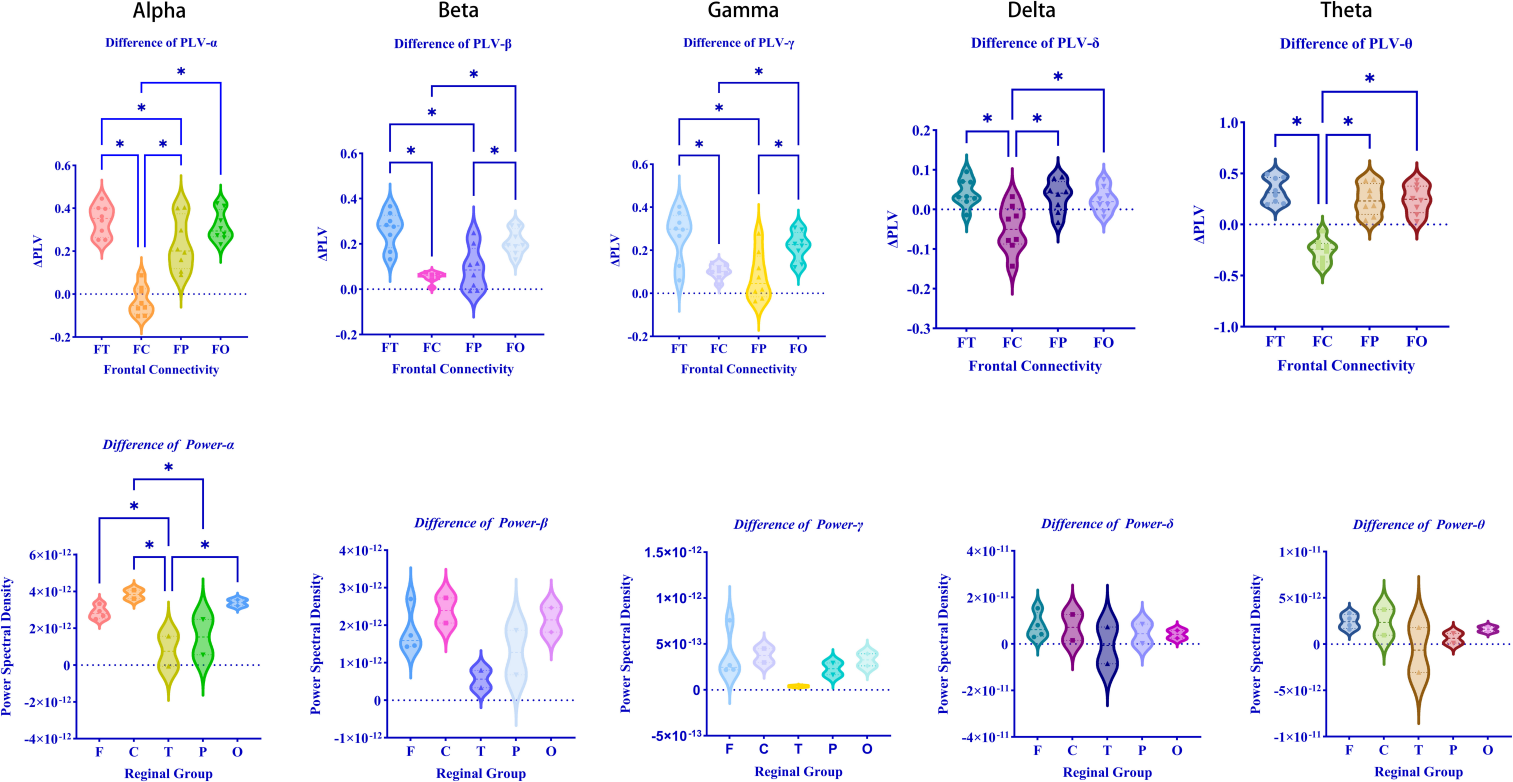
Interregional differences in stimulation induced ΔPLV and ΔAPSD across frequency bands. ΔPLV and ΔAPSD were calculated as E3 minus E0. The upper row shows interregional comparisons of ΔPLV between the frontal region and other cortical regions. The lower row shows interregional comparisons of ΔAPSD across predefined cortical regions. F, frontal (Fp1, F3, F7, Fz); C, central (C3, Cz); T, temporal (T3, T5); P, parietal (P3, Pz); O, occipital (O1, Oz). F-C, frontal-central; F-T, frontal-temporal; F- P, frontal-parietal; F-O, frontal-occipital. The symbol * indicates that in pairwise comparisons of multiple study groups, the differences in indicators between groups are statistically significant (*P* < 0.05).

Further analysis showed that stimulation related EEG changes were both time dependent and region specific. The temporal analyses showed progressive enhancement of frontotemporal PLV and frontal APSD over the stimulation period, particularly within the theta to alpha bands (Supplementary Figures 4, 5). Interregional analysis of stimulation induced changes revealed marked spatial heterogeneity across frequency bands (Figure 4). Frontotemporal ΔPLV was greater than frontocentral ΔPLV in most frequency bands, whereas ΔAPSD differences were most pronounced between frontal and temporal regions. Proportional contrast analysis further supported this spatially non-uniform pattern, showing that frontal-temporal contrasts contributed prominently to the overall interregional differences in stimulation induced EEG changes (Supplementary Figure 6).

### 3.4 TMS and clinical scales assessments

Figures 2C–E summarize the longitudinal clinical changes after selection of 80 Hz stimulation. Baseline refers to the pre-SCS assessment, after long-term SCS refers to the assessment performed after completion of the 21-day continuous 80 Hz stimulation period, and 6 months after discharge refers to the outpatient follow-up assessment. After completion of the 3-week stimulation, the latency of TMS-MEP was significantly shortened following SCS (*P* = 0.007). While no remarkable improvement in MEP amplitude was observed, the post-stimulation variabilities was markedly reduced (Table 1). In parallel, the CRS-R score increased to 10, while spasticity in both the upper and lower limbs improved substantially, with the MAS decreasing to 1 + and the HATS to 1 (Figure 2).

### 3.5 Follow-up after discharge

At 6 months after discharge, neurological follow-up assessment was performed. The CRS-R score increased to 14, corresponding to minimally conscious state plus. The patient was able to perform simple calculations (Supplementary Video). For motor function, MAS scores were 1 for the biceps brachii and adductor femoris and 1 + for the other assessed muscles. HATS remained at 1 point (Figure 2). The daily duration of spasticity decreased to approximately 2 h. No procedure or stimulation related complications were observed during hospitalization or follow-up.

## 4 Discussion

This case suggests that SCS may be associated with concurrent improvement in consciousness and spasticity in a patient with prolonged DoC following severe TBI. Frequency-sweep testing identified 80 Hz as the most favorable parameter for tone reduction in this patient. During the 21-day stimulation period, clinical improvements were accompanied by reduced sEMG activity, shortened TMS-MEP latency, enhanced left frontotemporal connectivity, and changes in regional spectral power.

The ability to integrate information across cortical regions may be a prerequisite for consciousness (Albantakis et al., 2023). APSD reflects regional spectral power of cortical electrical activity, whereas PLV estimates the synchrony of neural activity between regions. We considered the frontal lobe as the core of regional connectivity given the critical role that thalamic projections to the frontal lobe play in consciousness (Edlow et al., 2021). The results revealed pronounced ΔPLV differences between frontotemporal and frontocentral regions across all five frequency bands, accompanied by significant ΔAPSD variations in frontotemporal areas. In this single case, prolonged 80 Hz SCS was accompanied by reduced muscle tone, increased frontotemporal coupling, and changes in regional spectral power. These observations may suggest a potential association between SCS-induced clinical improvement and frontotemporal network changes.

Patients with unresponsive wakefulness syndrome exhibit fixed functional connectivity in the frontotemporal parietal network, whereas those in minimally conscious state retain partial dynamic reorganization capacity. Supporting this, Vorobyev et al. (2023) demonstrated that SCS improved consciousness and spasticity, with fMRI revealing enhanced connectivity between the right anterior insula and key regions including the posterior cingulate cortex, frontal eye fields, and parieto-occipital areas. Mayorova L. A. et al. (2025) observed significant increases in functional connectivity between the left parietal sulcus cortex and left supraorbital gyrus, the motor area of the left middle temporal gyrus and dorsal attention network. These findings support stimulation-associated network reorganization rather than isolated regional activation. Our findings extended this evidence by showing SCS promoted global network activation and frontotemporal connectivity remodeling, aligning with the global neuronal workspace theory where energy redistribution may reflect recursive prefrontal-temporoparietal loops for sensory-motor integration (Panda et al., 2022). Similarly, SCS may influence salience and sensorimotor related network dynamics involved in perception-action integration (Wu et al., 2024).

Spasticity after severe brain injury should not be viewed solely as a spinal reflex phenomenon. It is increasingly understood as a network-level disturbance of supraspinal and spinal sensorimotor control, involving impaired interactions among cortical motor regions, premotor and supplementary motor regions, subcortical structures, brainstem descending pathways, and spinal interneuronal circuits (Thibaut et al., 2015; Sangari and Perez, 2019; Mayorova et al., 2023). Disruption of this hierarchical control may weaken corticospinal inhibition and enhance corticoreticulospinal or reticulospinal facilitation, thereby increasing spinal reflex gain (Sangari and Perez, 2019). Within this framework, SCS may reduce spasticity through both segmental and supraspinal mechanisms. At the spinal level, activation of dorsal root and sensory afferent pathways may modulate interneuronal excitability and motoneuron recruitment (Formento et al., 2018; Greiner et al., 2021). At the supraspinal level, repeated spinal afferent input may reshape sensorimotor network dynamics and descending motor control (Moens et al., 2012; Mayorova et al., 2023). Accordingly, the reduced MAS and HATS scores, decreased sEMG activity, and shortened TMS-MEP latency observed in this case may reflect partial rebalancing of cortical–brainstem–spinal sensorimotor loops.

The concurrent improvement in consciousness and spasticity may arise from partially overlapping brain–spinal mechanisms. Arousal related systems and descending motor control pathways interact through the brainstem reticular formation, thalamocortical projections, frontal–sensorimotor networks, and ascending somatosensory afferents (Schiff, 2010; Formento et al., 2018; Edlow et al., 2021). Severe TBI may therefore simultaneously impair consciousness-related network integration and supraspinal regulation of spinal sensorimotor circuits. SCS may provide structured ascending somatosensory and proprioceptive input to brainstem and thalamocortical networks while also modulating spinal reflex excitability and descending motor control (Moens et al., 2012; Formento et al., 2018). In this context, enhanced frontotemporal EEG connectivity and spectral power may reflect facilitation of arousal-related cortical networks. This consciousness–motor interaction remains exploratory and requires validation using synchronized EEG–sEMG, neuroimaging, and longitudinal behavioral assessments.

We also observed an asymmetric cross-limb response, with upper-limb passive movement after SCS associated with a greater reduction in lower-limb spasticity than the converse response. Prior studies suggest functional coupling between upper- and lower-limb sensorimotor networks and modulation of lower-limb reflex excitability by arm activity (Frigon et al., 2004; Zehr et al., 2016). However, in the absence of synchronized kinematic recordings, H-reflex testing, and simultaneous EEG–sEMG analysis during passive movement, the underlying mechanism remains undetermined. This observation should therefore be interpreted as exploratory.

This study has several limitations. First, the single case design precludes causal inference and limits generalizability. Second, MAS, CRS-R, EEG, and sEMG assessments were not acquired synchronously, which limited direct temporal coupling between clinical and electrophysiological changes. Third, although 6-month follow-up was available, longer term durability and parameter stability remain unknown. Finally, the mechanistic interpretation of consciousness-motor circuit modulation remains exploratory and requires validation in larger prospective studies with standardized multimodal assessments.

## 5 Conclusion

This case suggests that 80 Hz SCS was associated with concurrent improvement in spasticity and consciousness in a patient with prolonged DoC following severe TBI. These clinical changes were accompanied by electrophysiological findings, including reduced sEMG activity, shortened MEP latencies, and enhanced frontotemporal functional connectivity. These findings provided preliminary evidence for the therapeutic potential of SCS in addressing the intertwined motor and arousal deficits in this challenging patient population and supported further investigation into the neural correlates associated with its dual modulatory effects.

## Data Availability

The data that support the findings of this study are available from the corresponding author upon reasonable request.

## Supplementary material

Supplementary Video This video shows pre- and postoperative evaluation of a patient with disorder of consciousness and spasticity undergoing spinal cord stimulation. Preoperative assessments include the CRS-R for consciousness and the MAS for upper and lower limb muscle tone. Postoperative reassessments reveal notable improvements in both CRS-R scores and MAS grades. Follow-up clips further demonstrate sustained functional gains in daily activities such as eating and cognitive tasks like arithmetic calculation.
