# Supplementary material for: Spinal cord stimulation for concurrent improvement of consciousness and spasticity following severe traumatic brain injury: a case study
Source: Front Neurosci. 2026 Jul 28;20:1866882. doi: 10.3389/fnins.2026.1866882 (PMC13458046; doi:10.3389/fnins.2026.1866882)
Supplement: Supplementary file 1 [file Data_Sheet_1.docx]

**Supplementary Materials**

**Supplementary figures.**

**Figure S1.** The brain functional connectivity reflected by PLV at different time points of long-term stimulation.

A. Baseline before SCS treatment.

B. The first day following the initiation of long-term stimulation

C. The 10th day following the initiation of long-term stimulation.

D. The 21th day following the initiation of long-term stimulation.

**Figure S2.** The curve of APSD alterations at different time points of long-term stimulation in different regions.

**Figure S3.** Heatmap of power under diverse frequency bands at different time points of long-term stimulation.

A. Alterations in brain power heatmap from day 1 to day 21 in alpha frequency band.

B. Alterations in brain power heatmap from day 1 to day 21 in beta frequency band.

C. Alterations in brain power heatmap from day 1 to day 21 in delta frequency band.

D. Alterations in brain power heatmap from day 1 to day 21 in gamma frequency band.

E. Alterations in brain power heatmap from day 1 to day 21 in theta frequency band.

**Figure S4.** The differences in PLV of functional connectivity between the frontal lobe and other brain regions across different frequency bands at various long-term stimulation time points.

**Figure S5.** The differences in APSD between the frontal lobe and other brain regions across different frequency bands at various long-term stimulation time points.

**Figure S6.** The proportion of ΔAPSD difference between frontal and other lobes and ΔPLV difference between frontal and other lobes across diverse frequency bands during long-term stimulation period.

**Figure S1.** The brain functional connectivity reflected by PLV at different time points of long-term stimulation.

A. Baseline before SCS treatment.

B. The first day following the initiation of long-term stimulation

C. The 10th day following the initiation of long-term stimulation.

D. The 21th day following the initiation of long-term stimulation.

**Figure S2.** The curve of APSD alterations at different time points of long-term stimulation in different regions.

**Figure S3.** Heatmap of power under diverse frequency bands at different time points of long-term stimulation.

A. Alterations in brain power heatmap from day 1 to day 21 in alpha frequency band.

B. Alterations in brain power heatmap from day 1 to day 21 in beta frequency band.

C. Alterations in brain power heatmap from day 1 to day 21 in delta frequency band.

D. Alterations in brain power heatmap from day 1 to day 21 in gamma frequency band.

E. Alterations in brain power heatmap from day 1 to day 21 in theta frequency band.

**Figure S4.** The differences in PLV of functional connectivity between the frontal lobe and other brain regions across different frequency bands at various long-term stimulation time points.

**Figure S5.** The differences in APSD between the frontal lobe and other brain regions across different frequency bands at various long-term stimulation time points.

**Figure S6.** The proportion of ΔAPSD difference between frontal and other lobes and ΔPLV difference between frontal and other lobes across diverse frequency bands during long-term stimulation period.

**Supplementary Video**
